# Methods to model and predict the ViewRay treatment deliveries to aid patient scheduling and treatment planning

**DOI:** 10.1120/jacmp.v17i2.5907

**Published:** 2016-03-08

**Authors:** Shi Liu, Yu Wu, H. Omar Wooten, Olga Green, Brent Archer, Harold Li, Deshan Yang

**Affiliations:** ^1^ Department of Radiation Oncology School of Medicine Washington University in St. Louis St. Louis MO; ^2^ ViewRay Incorporated Cleveland OH USA

**Keywords:** ViewRay, MR‐IGRT, statistical analysis, treatment delivery time, plan complexity

## Abstract

A software tool is developed, given a new treatment plan, to predict treatment delivery time for radiation therapy (RT) treatments of patients on ViewRay magnetic resonance image‐guided radiation therapy (MR‐IGRT) delivery system. This tool is necessary for managing patient treatment scheduling in our clinic. The predicted treatment delivery time and the assessment of plan complexities could also be useful to aid treatment planning. A patient's total treatment delivery time, not including time required for localization, is modeled as the sum of four components: 1) the treatment initialization time; 2) the total beam‐on time; 3) the gantry rotation time; and 4) the multileaf collimator (MLC) motion time. Each of the four components is predicted separately. The total beam‐on time can be calculated using both the planned beam‐on time and the decay‐corrected dose rate. To predict the remain‐ing components, we retrospectively analyzed the patient treatment delivery record files. The initialization time is demonstrated to be random since it depends on the final gantry angle of the previous treatment. Based on modeling the relationships between the gantry rotation angles and the corresponding rotation time, linear regression is applied to predict the gantry rotation time. The MLC motion time is calculated using the leaves delay modeling method and the leaf motion speed. A quantitative analysis was performed to understand the correlation between the total treatment time and the plan complexity. The proposed algorithm is able to predict the ViewRay treatment delivery time with the average prediction error 0.22 min or 1.82%, and the maximal prediction error 0.89 min or 7.88%. The analysis has shown the correlation between the plan modulation (PM) factor and the total treatment delivery time, as well as the treatment delivery duty cycle. A possibility has been identified to significantly reduce MLC motion time by optimizing the positions of closed MLC pairs. The accuracy of the proposed prediction algorithm is sufficient to support patient treatment appointment scheduling. This developed software tool is currently applied in use on a daily basis in our clinic, and could also be used as an important indicator for treatment plan complexity.

PACS number(s): 87.55.N

## I. INTRODUCTION

Cancer is one of the leading causes of death throughout the entire world. In the US, half of men and one‐third of women will develop cancers during their lifetimes and about half of them will receive radiation treatments.^1^, ^2^, ^3^ RT cancer treatments have developed from relatively simple processes into very complex procedures during the past two decades.^(4)^ The ViewRay System (VRS) (i.e., MRIdian system;ViewRay Inc., Oakwood Village, OH) is an integrated magnetic resonance (MR) image‐guided radiation therapy system designed to provide simultaneous MR imaging (MRI) and external‐beam radiation therapy (EBRT) treatment.^(5)^ The first commercial ViewRay system was installed in our institution.^(6)^ It has three cobalt‐60 treatment heads, 120° apart, and each head provides a nominal dose rate of 1.85 Gy / min at isocenter.^7^, ^8^, ^9^

**Figure 1:**
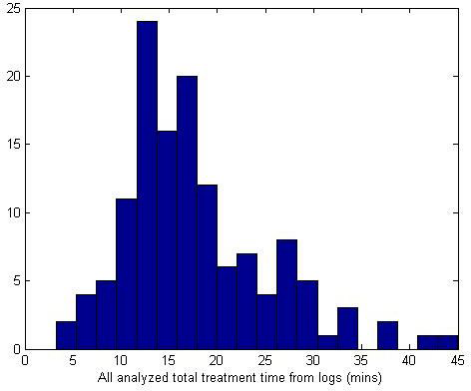
A histogram of the total treatment delivery time in minutes for patients treated in 2014.

Generally, the patient treatment time using ViewRay system consists of patient setup time, image acquisition time, image registration time, treatment delivery time, and finishing‐off time. The daily image acquisition time includes the time for pilot scanning, reviewing pilot, and high resolution scanning, and the “finishing‐off” time includes the time for finishing the treatment, submitting delivery record to MOSAIQ (IMPAC Medical Systems Inc., Sunnyvale, CA), and helping patient to exit the room. Based on the statistics recorded at our clinic, Table 1 lists the calculated averaged values and standard deviations of each time. The main motivation of this paper is to predict the total treatment delivery time at the completion of or during treatment planning in order to effectively schedule patient treatment appointments. VRS total treatment deliver time is a complex function of source decay, the order of beam deliveries, the gantry and MLC motions, and the final gantry position of the previous delivery. It is very difficult to accurately predict the total treatment time for the following reasons: (i) According to our clinical delivery records, the total treatment delivery time varies from approximately 3.28 min to 44.98 min, shown in Fig. 1, weakly correlated to the prescription, and the total numbers of beams and segments (a total 120 treatment delivery log files were used and analyzed). (ii) There are three cobalt‐60 treatment heads to allow simultaneous beam delivery. The total beam‐on time attributing to the total treatment time is decided by the last finished beam at one of three treatment heads during each gantry position simultaneous delivery. (iii) Cobalt‐60 sources decay. (iv) The VRS reorders the planned gantry positions according to the current gantry angle left from the previous patient treatment delivery so that VRS rotates its gantry only in one direction to finish the whole treatment without rotating back and forth. (v) The time spent for initialization operations before the first beam‐on depends on various random situations.

**Table 1:** Mean values and standard deviations (SDs) of the patient setup time, imaging and image registration time, treatment delivery time, and finishing‐off time (in min)

*Patient Setup*	*Imaging*	*Image Registration*	*Treatment Delivery*	*Finishing‐off*
4.95±2.78	6.75±1.66	4.82±2.31	15.19±6.54	2.89±2.06

Due to these complex reasons, in this paper we aim to: 1) systematically model the VRS treatment delivery, 2) statistically learn the unknown modeling parameters and constants from the recorded delivery log files, 3) predict the total treatment delivery time with acceptable accuracy based on the model after verification, and 4) aid and optimize the plan complexity using the predicted treatment time. The accuracy and feasibility of this prediction tool have been demonstrated to be a great improvement against a previous developed Microsoft Excel‐based prediction tool in our clinic. In particular, this Excel‐based tool does not support the updated source strengths after a recent cobalt source exchange, while our method considers the source decay carefully, which makes it more advantageous. Furthermore, the proposed method also indicates the treatment plan complexity, if necessary, to suggest that the treatment plan shall be improved to reduce the total treatment delivery time. This could be useful for future optimization study on the ViewRay treatment planning stage.

There are few similar studies reported in the literature. The most relevant published studies were about MLC leaf sequence optimization for step‐and‐shoot IMRT plans in the treatment planning stage.^10^, ^11^, ^12^, ^13^ Comparisons of dynamic and segmental MLCs (i.e., DMLC and SMLC, respectively), were reported and discussed involving total delivery time for the purpose of deciding which method to use.^(14)^ The proposed work shares similar mathematics as used in previous works in computation of MLC leaf motion time from the MLC motion distance and motion speed. While considerations of gantry rotation time and treatment initialization time are important additional components in this work, the most important contributions of this work are about the MRIdian‐specific components, including considerations and modeling of source decay, the MLC motion overall and interleaf delay times, and simultaneous beam deliveries with three treatment heads.

## II. MATERIALS AND METHODS

### A. The ViewRay treatment delivery procedure

**Figure 2:**
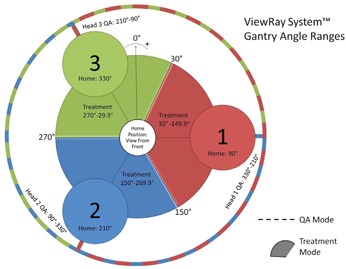
The ViewRay System gantry angle ranges for each treatment head. Head 1 has the range of 30° to 150°; Head 2 has the range of 150° to 270°; Head 3 has the range of 270° to 30°.

The VRS delivery system has three cobalt‐60 treatment heads, 120° apart, with each providing a nominal dose rate, 1.85 Gy / min, at new‐strength installation, however decaying at a half‐life time of 5.25 yrs. The three heads together provide a total dose rate comparable to that of conventional linear accelerator (linac) using simultaneous delivery. Treatment plans are created in the ViewRay treatment planning system (TPS). Each plan contains multiple treatment beam groups (i.e., gantry positions) with each containing one to three beams. Beams belonging to the same beam groups have gantry angles 120° apart and therefore could be delivered simultaneously by three treatment heads. Each beam contains one or multiple segments. Each segment is defined by a MLC formed beam aperture and a beam‐on time. There are, totally, 60 MLC leaves in 30 pairs. As one can see in Fig. 2, besides that three treatment heads are at 120° apart, each head also has a limited angle range.

On the treatment day, after patient is set up on the couch table, VRS will rearrange the order of the beam groups so that they will be delivered as the gantry goes either clockwise or counterclockwise. This is to avoid rotating the gantry back and forth and therefore to minimize the total treatment delivery time. The rotation direction is selected so that the starting gantry angle is as close as possible to the current gantry angle left from the last treatment delivery. VRS then rotates the gantry to the starting position, and moves the MLC leaves from open‐field positions to the planned MLC positions of the first beam segments.

In VRS, beams are delivered in the step‐and‐shoot way. Radiation will be turned on for delivering one beam segment at a time. Between segments, the radiation will be turned off (the cobalt source will be moved to the off position) and the MLC leaves will move to the next position. It is important to note that the MLC leaves are not moving simultaneously due to the limitations in the software and hardware systems. The MLC control system can send only one motion command message to one leaf at a time. This leads to an interleaf motion delay. First, the MLC control system will determine which side of the leaves pairs will be moving away from the opposite side and start moving leaves on this side one by one. Once those leaves on the beginning side are all moving then the control system will move the leaves on the other side one by one. Beams belonging to a beam group are delivered simultaneously and independently by the three beam heads. After deliveries of one beam group, the VRS rotates the gantry to the next position. While rotating, MLC leaves in each head move to the next planned positions. After the gantry reaches the next position, the next group of beams will be simultaneously delivered in the same step‐and‐shoot way.

### B. Prediction method

#### B.1 The scheme of our algorithm

**Figure 3:**
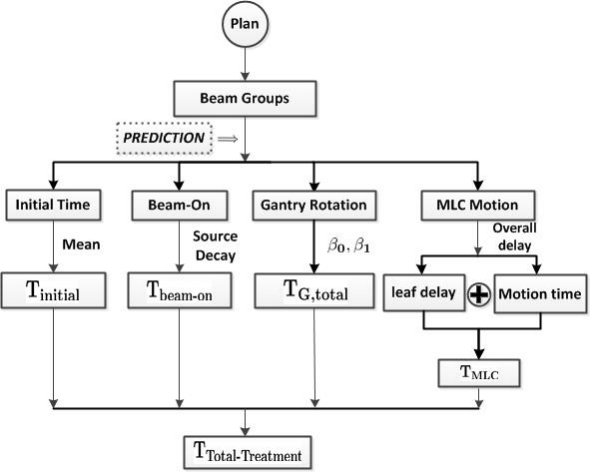
A schematic illustration of the treatment time prediction procedure.

With the understanding of the treatment delivery procedure, we model the total treatment delivery time in four components: 1) the treatment initialization time; 2) the total beam‐on time; 3) the gantry rotation time; and 4) the MLC motion time. The gantry rotation time is calculated per beam group. The beam‐on time and MLC motion time are calculated per beam segment for each beam, and then processed per beam group. Figure 3 illustrates our prediction algorithm, which requires only the treatment plan data file, and each time component is predicted separately.

In this study, the patient treatment delivery log files and the treatment plan data files are collected from the beam delivery and the planning stages respectively.^(4)^ A total 120 log files were used and formed into a log file database. Computer codes were developed in MATLAB (The MathWorks, Inc., Natick, MA) to process these data files.

#### B.2 Predicting the initialization time

The initialization time includes the time required for the gantry to rotate to the first position. This time term is rather random since it depends on the last gantry position of the last finished treatment. To estimate this component, we use the averaged value of the treatment initialization time $\left(T_{\text{initial}\_\text{log}}\right)$ extracted from the treatment delivery log file database as:

(1) $$T_{Initial}=mean(T_{initial\_\text{log}})$$

#### B.3 Calculating the beam‐on time

In order to predict the total beam‐on time, the actual dose rate must be determined. In VRS, the dose rates of the treatment heads decrease at half‐life (HL) time of 5.25 yrs and can be calculated as:

(2) $$DR(j)=DR_{ref}(j)\times {\left(\frac{1}{2}\right)}^{ndays/HL},j=1,2,3$$

where *DR(j)* denotes the dose rate of the treatment head *j* on the treatment day, $DR_{\text{ref}}(j)$ denotes the reference dose rate of the treatment head *j* on the source strength calibration date (i.e., the reference date), and *ndays* represents the number of days between the treatment delivery day and the reference date.

The beam‐on time for each beam can be calculated as:

(3) $$T_{beam-on}(b)=\frac{T_{planned-beam-on}(b)\times 1.85\;Gy/\text{min}}{DR(j)}$$

where $T_{\text{beam}-\text{on}}(b)$ is the calculated beam‐on time for all segments of the beam *b* with the treatment head *j*, and $T_{\text{planned}-\text{beam}-\text{on}}(b)$ is the planned beam‐on time for the same beam based on the nominal dose rate 1.85 Gy / min used in the treatment planning stage.

(3) $$T_{beam-on}(b)=\frac{T_{planned-beam-on}(b)\times 1.85\;Gy/\text{min}}{DR(j)}$$

#### B.4 Predicting the gantry rotation time

The prediction for the gantry rotation time is achieved using linear regression. The relationship between the rotation angle (i.e., the gantry angle difference between a beam group and the next beam group) and the corresponding rotation time can be modeled with a linear regression model:

(4) $$T_{G}(g)=\beta_{0}+\beta_{1}\cdot \theta_{diff}(g)$$

where $T_{G}(g)$ and $\theta_{\text{diff}}(g)$ are the gantry rotation time and the gantry angle difference between the beam groups *g* and g+1, respectively, and $\beta_{0}$ and $\beta_{1}$ are the linear regression fitting parameters.

**Figure 4:**
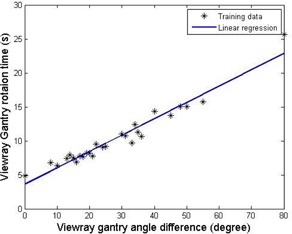
Linear regression on gantry rotation time. The markers * represent the data points extracted from the log file database. The straight line is the linear fitting. The coefficient of determination (i.e., $R^{2}$) is 0.9710.

The data points obtained from the log file database were used to compute $\beta_{0}$ and $\beta_{1}$. The gantry rotation time $T_{G}(g)$ in the previous treatment deliveries were obtained by computing the time between the beam‐off time of a beam group and the beam‐on time of the next gantry position. Figure 4 shows that the gantry angle differences (x‐axis) and the gantry rotation times (y‐axis) fit well linearly. The coefficient of determination (i.e., $R^{2}$), described as one measurement of goodness of the linear regression, is calculated to be 0.9710, which demonstrate that our linear regression predicts with the accuracy 97.1% of the variance on the gantry rotation time.

Finally, to predict the total gantry rotation time $T_{G,\text{total}}$ for the given plan, 1) the beam groups are reordered so that the gantry angle positions are in the ascending/descending order, 2) $T_{G,\text{total}}$ is calculated by summing all the predicted gantry rotation time in between the reordered beam groups:

(5) $$T_{G,total}=\sum_{g=1}^{Ng-1}T_{G}(g)$$

where $N_{g}$ is the total number of beam groups and there are a total $N_{g}-1$ gantry rotations.

It is worth noting that the beam groups in treatment deliveries would be reordered by the treatment machine in either ascending or descending order to ensure that the gantry rotates always in one direction. The gantry rotation direction will not affect the predicted gantry rotation time because the angle differences between the beam groups do not change regardless of the gantry rotation direction.

#### B.5 Predicting the MLC motion time

The MLC motion time, defined as the time for the MLC leaves to move from the position of a beam segment to the position of the next beam segment, is complicated and dependent on multiple factors including the control system delays, the MLC leaf motion speed, the MLC leaf positions and the numbers of MLC leaves in motion. Based on our communication with ViewRay engineers and our own observations of the MLC motions displayed on the treatment machine console during the treatment deliveries, we model the MLC motion using the following three terms:

1. The overall delay $T_{\text{MLC}-\text{overall}-\text{delay}}$: defined as a) the delay after beam‐off for the previous segment and before MLC leaves start to move toward the next positions, and b) the delay after the MLC leaves arrive the target position and before the beam‐on for the next beam segment. This overall delay is due to both the cobalt sources’ end effect and the delays in VRS control system.
2. The interleaf delay $T_{\text{inter}-\text{leaf}-\text{delay}}$: defined as the time delay between one leaf and the next leaf starting to move. The delay is due to the MLC motion control system's being only able to send the command to one leaf at a time. There are two additional important notes: a) the MLC leaves on one side are firstly moving away from the opposite leaves one by one, and only after all the MLC leaves on one side are moving will the leaves on the opposite side start to move; b) only the MLC leaves changing positions between beam segments are involved. The MLC leaves that are not changing their positions are skipped by the inter‐MLC‐leaf delay process and therefore ignored by the MLC motion prediction calculation.
3. The leaf motion time $T_{\text{leaf}-\text{motion}}$: defined as the time for a leaf to move from the current position to the next position.

Given these three time terms, the motion time for a single MLC leaf is predicted as:

(6) $$T_{MLC,i}=T_{mlc-overall-delay}+i\cdot T_{\text{int}er-leaf-delay}+\frac{d_{motion,i}}{v_{leaf}}$$

where *i* is the MLC leaf number and i=1 to 60, $d_{\text{motion},i}$ is the motion distance (cm) of the leaf *i*, and $v_{\text{leaf}}$ is the MLC leaf motion speed (cm/s).

The MLC motion time for the beam *b* and segment s, denoted as $T_{\text{MLC}}(b,s)$, can be obtained by calculating the maximum values of $T_{\text{MLC},i}$ for all 60 MLC leaves for this beam segment, ignoring the leaves not moving, as:

(7) $$T_{MLC}(b,s)=\text{max}(T_{MLC,i}),i=1\;to\;60$$

For each beam, the MLC motion time can be calculated by summing up the MLC motion time per segment:

(8) $$T_{MLC}(b)=\sum_{s=1}^{N_{s}-1}T_{MLC}(b,s)$$

where $N_{s}$ is the number of beam segments for this beam.

It is important to note that 1) there are only $N_{s}-1$ MLC motions between the $N_{s}$ beam segments, and 2), as we have observed at the treatment machine console, the MLC apertures of the first beam segments are formed during the gantry rotation and before the gantry rotation finishes. Thus the time for MLC leaves moving to the first beam segment positions is already covered in the gantry rotation time.

The MLC motion model parameters were established by analyzing the screen capture videos recorded on the treatment machine console during treatment deliveries. $T_{\text{MLC}-\text{overall}-\text{delay}}$ was estimated to be approximately 2.2 s. $T_{\text{inter}-\text{leaf}-\text{delay}}$ was estimated to be 0.034 s. The average speed of the leaf motion was estimated to be approximately 2.2 cm / s.

#### B.6 Predicting the total treatment delivery time

Due to simultaneous deliveries with three treatment heads for each beam group, in order to obtain the total treatment time, it is important to determine which beam within the beam group finishes last. The last finished beam in the beam group *g*, denoted as $b_{g}$, is determined by the maximum sum of the beam‐on time and beam MLC motion time among (up to) three beams in the same beam group. The total treatment delivery time can be obtained as follows:

(9) $$T_{total}=T_{Initial}+T_{G,total}+\sum_{g=1}^{Ng}T_{beam-on}(b_{g})+\sum_{g=1}^{Ng}T_{MLC}(b_{g})$$

where $N_{g}$ is the total number of beam groups.

#### B.7 Verification of the prediction models with simulation

Because the prediction models we have developed in this study, especially the MLC motion model, are relatively complicated, it is very important to verify these models before applying them into prediction. Therefore we developed a computer program in MATLAB to graphically simulate the entire treatment delivery. This simulated graphic presentation was visually compared to the recorded videos on the treatment machine console to qualitatively verify the prediction models.

**Figure 5:**
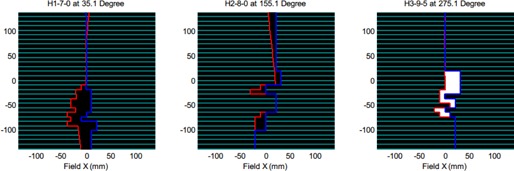
A screenshot of a simulated treatment video for a particular patient. The title of each subfigure describes the treatment delivery status information for the corresponding treatment head index, beam number, segment number, and gantry angle.

Figure 5 shows a snapshot of one simulated treatment delivery. The MLC motions in three treatment heads are illustrated separately and independently in three subfigures. The title of each subfigure describes the treatment delivery status information for the corresponding treatment head index, beam number, segment number, and gantry angle. In this screen capture, beams are off at head 1 and 2, and the MLC leaves are moving towards to the next positions (the segment indices are arbitrarily set to zero in this case). Treatment head 3 is currently delivering the ninth beam and its fifth segment. The MLC aperture in white indicates that beam is on.

## III. RESULTS

In our simulated prediction model of the VRS treatment time, various modeling parameters were proposed and applied for predictions, which are listed in Table 2 with corresponding values. A total of 120 log files and their corresponding plan files were used to verify the overall prediction accuracy. The mean values and the standard deviations of each time component and total delivery time, as well as the prediction errors, for all the analyzed log files, are listed in Table 3. The overall treatment delivery time prediction error is 0.21±0.28 minutes.

Table 4 presents a comparison of actual and predicted treatment time for one specific patient example. By calculating the difference between the treatment delivery times predicted based on the patient treatment plan data file and the actual treatment delivery time extracted from the treatment delivery log file for the same treatment plan, the prediction errors are 0.21±0.28 minutes. Significantly, the mean error percentage, i.e., the percentage of the absolute prediction, is 1.82%, which is far less than the prediction error using the Microsoft Excel‐based predictor (15.75%). The max prediction error is 0.49 minutes and its corresponding maximum error percentage is equal to 7.88%, which is also a great improvement from the maximum error seen using the previous Excel‐based predictor (24.85%).

It is worth noting that this prediction error includes the errors arising from a random factor, such as the initialization time. If the initialization time is eliminated from the total delivery time, the maximum prediction error of our method is 0.36 minutes.

**Table 2:** Summary of the prediction parameters used in our simulation model

*Modeling Parameters*	*Value*
$T_{\text{initial}}$	27.7 (s)
$\left[\beta_{0},\beta_{1}\right]$	(3.61, 0.24)
$T_{\text{MLC}-\text{overall}-\text{delay}}$	2.2 (s)
$T_{\text{inter}-\text{leaf}-\text{delay}}$	0.034 (s)
$v_{\text{leaf}}$	2.1 (cm/s)

**Table 3:** Mean values and SDs (min) of four treatment time components

*Components*	*Mean*	*SD*	*Prediction Error* (mean±SD)
$T_{\text{initial}}$	0.463	0.149	0.11±0.15
$T_{\text{beam}-\text{on}}$	10.085	7.098	−0.03±0.06
$T_{G,\text{total}}$	0.910	0.589	0.11±0.19
$T_{\text{MLC}}$	2.210	2.025	−0.29±0.24
$T_{\text{total}}$	15.19	6.54	0.21±0.28

**Table 4:** An illustration of a patient's treatment time prediction result (in min) compared to the actual treatment delivery time from the corresponding recorded log file

*Components*	*Predicted Treatment Time*	*Actual Treatment Time*	*Prediction Error*
$T_{\text{initial}}$	0.5757	0.4153	0.1604
$T_{\text{beam}-\text{on}}$	8.1655	8.1652	0.0003
$T_{G,\text{total}}$	0.6217	0.6251	−0.0034
$T_{\text{MLC}}$	5.9433	6.0965	−0.1532
$T_{\text{total}}$	15.3062	15.3021	0.0041

**Figure 6:**
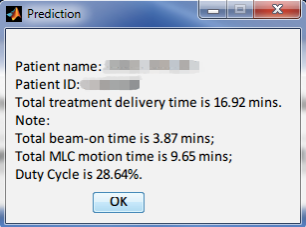
Screenshot of the treatment delivery time prediction tool.

Based on the proposed prediction algorithm, we have developed a software tool using MATLAB for our dosimetrists and physicists to use for scheduling patient treatment appointments. It requires only the treatment plan data file as the input. Screenshots of this program are presented in Fig. 6. One can see that the expected total treatment time is given with important time components listed and duty cycle calculated (defined in Section IV.B). This tool is currently used daily in the clinic.

## IV. DISCUSSION

### A. Effects of moving the closed MLC pairs on the total MLC motion time

**Figure 7:**
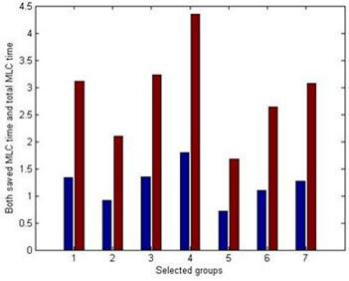
Examples of possible saved MLC time compared its corresponding total MLC motion time. The bar with slash ‘\’ pattern on the left of each group represents the possible saved MLC time in minutes and the bar with star ‘*’ pattern on the right of each group represents its total MLC motion time recorded in the actual treatment deliveries.

Use of the MLC‐shaped beam segments in IMRT requires accurate modeling of the MLC leaf transmission and interleaf leakage. Based on our observations from the recorded treatment delivery videos and the MLC position values in the treatment plans, VRS purposely moves the closed MLC leaf pairs between the beam segments even though these closed MLC leaf pairs do not contribute to the beam aperture definition. The positions of the closed MLC leaf pairs are planned by the TPS in order to avoid the leakage dose of MLC junctions at the fixed position. However, if these closed leaf pairs remained still while only those leaves forming the aperture moved, total MLC motion time could be significantly reduced. While the MLC leaf junction leakage still needs to be carefully considered, one alternative method could be moving these closed MLC junctions per treatment fraction instead of per beam segment. We analyzed the time taken to move these closed pairs for each treatment in the log file database. If the closed MLC leaf pairs do not move, the average saved MLC motion time percentage at each gantry position is 33.40%, shown in Fig. 7.

### B. Effects of plan complexity

We have observed that the treatment delivery time for individual treatment plans vary significantly, even among the plans treating the same treatment sites with similar prescription doses. We have therefore performed an analysis on plan complexity in order to understand the relationship between plan complexity and treatment delivery time, with the intention of aiding the treatment planning process.

The complexity of treatment plans arises from beam modulation (i.e., some plans require a large number of small and/or irregularly shaped beam segments). Therefore, we have analyzed a few important plan complexity metrics involving the beam modulation (BM) and the plan modulation (PM).^(15)^ BM can be described as the deviations of the MLC aperture shapes from a circle and BM indicates the extent of a large open field being broken into multiple small segments. PM is denoted as the plan averaged beam modulation obtained by averaging the BM values using the normalized beam‐on time (by 2 Gy nominal prescription dose) as weighing factors. Further details and definitions can be found in Du et al.^(15)^ The duty cycle and the total treatment time were found to correlate with BM and PM for all the treatment deliveries in the log files database. The duty cycle of each beam can be defined as the percentage of the total beam‐on time in each beam delivery, involving the sum of the beam‐on time and the MLC motion time. Similarly, the overall duty cycle of the plan is defined as the percentage of the total beam‐on time in the total treatment delivery time.

**Figure 8:**
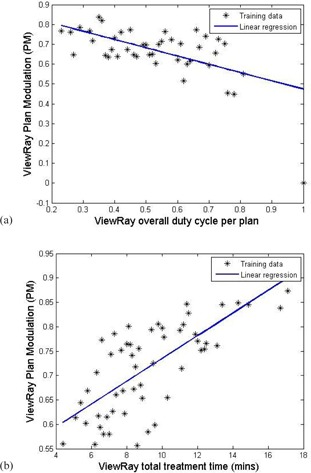
The relationships of (a) the overall plan duty cycle and the plan modulation (PM), (b) the predicted total treatment time (normalized to 2 Gy / fraction) and the PM, respectively.

Figure 8 shows the linear correlation between (a) the overall plan duty cycle and PM, and (b) the predicted total treatment delivery time and PM, respectively. We note that a single‐term exponential correlation method was also applied and similar trend was found to support the linear relationship between both the overall plan duty cycle and total treatment time versus the PM values. Only the dominant beam (last‐finished beam head) in each gantry position affects the total duty cycle and the total treatment time, and complexity shown only on such beams is investigated. As can be seen, the correlations indicate that increased complexity will lead to a decreased duty cycle, and more importantly result in a longer treatment delivery time.

Understanding of the plan complexity versus the treatment delivery time could be used in a few different ways.^(16)^ In particular, the treatment delivery time could be predicted using the proposed prediction algorithm and the predicted treatment time could be compared against the mean values of previous treatment plans of the same treatment site. If the predicted treatment time of the new plan is much longer than expected, the complexity on the dominant beams of this plan could be further optimized in order to reduce the treatment delivery time. If it is not practical to control the plan complexity on the individual beam, the overall plan complexity could be controlled.

## V. CONCLUSIONS

In this paper, we presented a technical note for the ViewRay treatment time prediction. By simulating the VRS treatment delivery, prediction models were developed for each treatment time component. The proposed method provides accurate treatment delivery time prediction. It is useful in scheduling patient treatment appointments, and for managing plan complexity at the treatment‐planning stage.

## ACKNOWLEDGMENTS

Research reported in this study was partially supported by the Agency for Healthcare Research and Quality (AHRQ) under award 1R01HS0222888, and partially supported by a research grant from ViewRay Incorporated. The content is solely the responsibility of the authors and does not necessarily represent the official views of the Agency of Healthcare Research and Quality.

## COPYRIGHT

This work is licensed under a Creative Commons Attribution 4.0 International License.
